# Transition to Widowhood: Trajectories of Depressive Symptomatology Among Japanese Older Adults

**DOI:** 10.1093/geroni/igab046.1099

**Published:** 2021-12-17

**Authors:** Masumi Iida, Shohei Okamoto, Ikuko Sugawara, Erika Kobayashi

**Affiliations:** 1 Arizona State University, Tempe, Arizona, United States; 2 Tokyo Metropolitan Institute of Gerontology, Itabashi, Tokyo, Japan; 3 Bunri University of Hospitality, Suginami-ku, Tokyo, Japan; 4 Tokyo Metropolitan Institute of Gerontology, Itabashi-ku, Tokyo, Japan

## Abstract

Spousal loss is one of the most consequential negative life events for the surviving partners. While there is abundant research on mental health and well-being of widows, most of these studies rely on the post-bereavement data. In this study, we use the data from the National Survey of Japanese Elderly (NSJE), which is a publicly available longitudinal data set collected from Japanese adults aged 60 years and older. The current study uses the first seven waves of data from 1987 to 2006, where participants were followed every three to four years. Using the NSJE advances our understanding of the bereavement process as it allows us to observe the levels and trajectories of depressive symptom before, during, and after the loss of their spouses. In our analyses, we selected 522 participants (average age at bereavement: 75.0 years; 27% male) who experienced spousal loss at some point during the seven waves. We examined the trajectories of depressive symptoms assessed using CES-D as these participants transition to widowhood. The results showed a small significant increase in depressive symptoms leading up to the time of the loss. There was also a significant increase in symptoms at the time of the loss, but we did not observe any decline in symptoms after the loss. In addition, we found that their age at bereavement significantly moderated the pattern, such that the increase in depressive symptoms at the time of the loss was attenuated for older participants. The implications of these findings will be discussed.

